# Relationship Between Physical Activity Frequency and Cardiovascular Risk Throughout the Life Cycle

**DOI:** 10.3390/jfmk11010091

**Published:** 2026-02-25

**Authors:** Oscar Araque, Luz Adriana Sánchez-Echeverri, Ivonne X. Cerón

**Affiliations:** 1 Departamento de Desarrollo Tecnológico, Project Medicine and Science, Universidad de Ibagué, Ibagué 730001, Colombia; 2 Departamento de Fisica, Facultad Ciencias, Universidad del Tolima, Barrio Santa Helena A.A. 546, Ibagué 730006299, Colombia; lasancheze@ut.edu.co; 3 Departamento de Producción y Sanidad Vegetal, Facultad Ingeniería Agronómica, Universidad del Tolima, Barrio Santa Helena A.A. 546, Ibagué 730006299, Colombia; ixcerons@ut.edu.co

**Keywords:** physical activity, cardiovascular health, hemodynamic stability, body composition, biochemical parameters

## Abstract

**Objectives**: Cardiovascular diseases (CVD) remain a leading cause of premature mortality globally, despite the proven efficacy of physical activity in reducing risks. This research aims to identify risk characteristics and characterise pathologies related to the onset of CVD in relation to physical activity levels. The study tests the hypothesis that adequate physical activity is associated with CVD-related events, while sedentary behaviour is a factor related to increased risk factors. **Methods:** A cross-sectional, observational, descriptive, and analytical study was conducted with 116 participants of both sexes (aged 16 to 77 years) in El Espinal, Tolima. Clinical, anthropometric, and biochemical assessments were performed, including blood pressure, Body Mass Index (BMI), visceral fat, and lipid profiles. Physical activity was self-reported and categorised as weekly, monthly, and occasional exercise. Descriptive and bivariate statistical analyses were performed. Quantitative variables were expressed as means and standard deviations. Qualitative variables were presented as absolute frequencies. Statistical interaction graphs were used to analyse the effects of age and exercise frequency on pulse pressure. **Results:** Weekly exercise was identified as a key modulator of hemodynamic stability; while BMI and visceral fat increased with age, pulse pressure remained stable (44.17–46.55 mmHg). In contrast, occasional exercise was linked to high cardiovascular vulnerability, with pulse pressure spiking to a critical 75.00 mmHg in elderly participants (77 years) and BMI reaching obesity levels (38.15 kg/m^2^). Monthly exercise showed high variability and progressive lipid profile deterioration, with total cholesterol reaching 282.00 mg/dL in late maturity. **Conclusions:** Regular weekly physical activity acts as a physiological buffer that dissociates chronological ageing from vascular damage. While weekly exercise maintains optimal hemodynamic and metabolic ranges, occasional or inconsistent activity fails to prevent critical increases in pulse pressure and arterial stiffness during senescence. These findings underscore the necessity of regular, rather than sporadic, exercise as a vital “medicine” for maintaining arterial integrity across the lifespan.

## 1. Introduction

Physical training (PT) and regular physical activity are fundamental tools in the prevention and treatment of cardiovascular disease (CVD) and are inversely associated with premature mortality. Exercise has been shown to reduce the risk of CVD mortality by 35% and all-cause mortality by 33%. Even a minimal intervention of 15 min of daily exercise can provide significant health benefits [1,2,3].

The positive impact of physical exercise on reducing risk factors and critical risk parameters for heart health has been identified. It is known that training significantly lowers blood pressure, achieving decreases of up to −8.3/−5.2 mmHg in hypertensive patients [4]. In addition, exercise and lifestyle change programmes can reduce the incidence of type 2 diabetes by 58% and improve glycaemic control by reducing glycosylated haemoglobin (HbA1c) by between 0.6% and 0.8% [5].

Physical activity and exercise in adults over 80 years of age is particularly beneficial because the prevalence of CVD exceeds 85% [6]. Physical activity mitigates oxidative stress and chronic inflammation linked to ageing. For this group, at least 150 min of moderate activity per week, such as brisk walking, is recommended [7]. Physical activity generates shear stress in the arteries, activating enzymes that release NO and mobilise progenitor cells to repair tissue [8]. These mechanisms improve flow-mediated dilation, reducing the risk of future cardiac events by up to 13%. In addition, performing aerobic exercise beforehand or interrupting prolonged periods of sedentary behaviour prevents acute endothelial dysfunction [9].

In patients with hypertension or diabetes, regular aerobic exercise lowers blood pressure and improves glucose control [10]. Vascular protection is effectively achieved regardless of the intensity or type of training applied. Thus, movement is consolidated as a vital medicine for arterial integrity [11].

Internally, exercise triggers repair and protection processes. In relation to vascular function, it improves the health of blood vessels by increasing the bioavailability of nitric oxide (NO) through the regulation of eNOS and the mobilisation of endothelial progenitor cells for tissue repair. Ref. [12] promotes cellular signalling by activating essential cascades such as IGF1/PI3K/AKT, which induce adaptive physiological cardiac hypertrophy and protect the myocardium [13]. Exercise regulates molecules such as non-coding RNAs like miR-222 [14], which are considered potential targets for new therapies against CVD.

Current cardiovascular health analysis highlights that endothelial dysfunction is a critical process that precedes the formation of atherosclerosis [15]. This condition is primarily defined by an imbalance in nitric oxide production and can be detected using methods such as flow-mediated dilation (FMD) [16,17], offering significant prognostic value in both primary and secondary prevention. To counteract this deterioration, cardiac rehabilitation based on aerobic exercise has proven to be an effective tool, as it improves the bioavailability of nitric oxide, increases endothelial progenitor cells, and reduces oxidative stress [18].

The clinical benefits of exercise are accurately reflected in improved flow-mediated dilation (FMD). The importance of this indicator is such that an increase in FMD can reduce cardiovascular risk by between 8% and 13% [19]. Conversely, it has been observed that a loss of just 1% in endothelial function increases the risk of cardiovascular events by 13% [20]. Given the negative impact of prolonged sedentary behaviour, it has been shown that performing a prior aerobic exercise session or interrupting the time spent sitting are effective tactics for preventing acute lower limb dysfunction [21].

With regard to metabolism, there are marked gender differences; men tend to accumulate more visceral fat [22], which increases the synthesis of very low-density lipoproteins (VLDL) and generates a more dangerous lipid profile (small, dense LDL). However, in women, high triglycerides represent an even stronger cardiovascular risk factor than in men [23]. To control these lipids, the use of statins is essential, reducing LDL cholesterol by between 20% and 40% by inhibiting the enzyme HMG-CoA reductase [24,25].

With regard to blood pressure control, it has been observed that this is the greatest risk factor in relation to CVD [26]. It has been found that a decrease of just 5 mmHg in systolic pressure significantly reduces the risk of cardiovascular events, both in patients with type 2 diabetes and in those without it [27]. Hypertension affects more than 31% of people in the development of heart disease [26]. Appropriate treatment can reduce the incidence of strokes by 36%.

Current scientific evidence positions physical exercise as a fundamental pillar in both the prevention and treatment of chronic non-communicable diseases (CNCDs) [28]. Its systematic implementation not only improves quality of life, but also significantly reduces morbidity and mortality associated with conditions such as hypertension, diabetes, and heart failure [29].

In hypertension, regular aerobic activity (5–7 days/week, >30 min) reduces systolic blood pressure by 5–8 mmHg. This benefit is linked to improved vasodilator function and the post-exercise hypotension effect [4]. With regard to diabetes, engaging in at least 150 min of moderate activity per week optimises insulin sensitivity and reduces pancreatic fat. [30]. Combined training (strength and aerobic) proves to be the most effective for controlling glycated haemoglobin [31].

For heart failure, exercise is a key recommendation that significantly reduces mortality and hospitalisations. In these patients, high-intensity interval training (HIIT) is often more effective than continuous training in increasing oxygen consumption [32]. However, prescribing must be individualised and supervised to ensure clinical safety [33].

Today, artificial intelligence (AI) has transformed cardiovascular prevention by driving precision medicine based on real-world data [34]. Unlike traditional methods, AI uses machine learning to identify hidden patterns, surpassing conventional risk scores and detecting subclinical diseases using tools such as automatic coronary calcium scoring [35]. In clinical practice, this technology optimises the management of hypertension and dyslipidaemia by predicting the response to drugs and facilitating the detection of familial hypercholesterolaemia [36]. In addition, AI supports decision-making through geospatial analysis of ‘hot spots’ and the use of chatbots such as EndeavourAI to offer personalised lifestyle guidance [37]. Continuous monitoring and integration of hospital data enable early warnings to be generated and significantly improve treatment adherence [38,39]. Together, these tools ensure timely interventions and optimised health trajectories for each individual.

In general, exercise reduces mortality from CVD by between 19% and 31%, and it has been observed that very high levels of activity (5 to 7 times the recommended amount) continue to be beneficial with no apparent upper limit. The integration of artificial intelligence (AI) into cardiac rehabilitation (CR) is redefining the management of coronary patients through a dynamic and highly personalised approach [40,41]. The use of machine learning algorithms, such as the XG Boost model, enables the automation of exercise prescriptions tailored to each individual, setting weekly step and calorie expenditure goals based on demographic data and the patient’s perception of exertion using the Borg scale [42]. Beyond physical training, technology facilitates comprehensive and qualitative monitoring [43].

From the above, it can be seen that one of the biggest obstacles to the prevention and treatment of CVD is low physical activity and low compliance rates among patients with prescribed exercise routines. The objective of this research is to identify risk characteristics and characterise pathologies related to the onset of CVD in relation to physical activity. Our hypothesis is that people who engage in adequate physical activity are associated with CVD-related events, while people who are primarily sedentary may present risk factors more frequently.

## 2. Materials and Methods

### 2.1. Research Subject

The study population consisted of community members residing in the city of El Espinal, Tolima, who participated voluntarily during the month of November 2025. Participants engaged in various activities as office workers, students, informal workers, and homemakers. The ages of the participants ranged from 16 to 77 years. The total number of participants was *n* = 116.

The diversity of the participants meant that the sample included young people with regular exercise routines and people who rarely exercised, as well as people with pre-existing conditions and/or CVD-related diseases. A confidentiality agreement was signed with the participants to protect the personal data obtained in this study.

### 2.2. Study Design

An observational, descriptive, and analytical cross-sectional study was conducted with an epidemiological–clinical approach, aimed at characterising the cardiometabolic profile and physical activity level of the participants, as well as exploring associations between anthropometric, biochemical, and lifestyle variables. Measurements were taken at a single point in time, without experimental intervention or treatment assignment.

#### 2.2.1. Population and Sample

The reference population consisted of individuals who voluntarily agreed to participate in the study. The sample corresponded to a non-probability convenience sample, comprising participants who met the inclusion criteria and agreed to sign the informed consent form.

Inclusion criteria: individuals ≥16 years of age, voluntary acceptance and signing of the informed consent form, and willingness to undergo anthropometric measurements and blood sampling. The analysis is performed by age range, as illustrated in Table 1.

Exclusion criteria: incomplete information on the variables of interest and inability to take anthropometric measurements or blood samples.

#### 2.2.2. Procedure and Data Collection

The information collection process was carried out in a standardised and sequential manner:

Ethical and administrative aspects: Prior to any procedure, each participant was informed of the purpose of the study, the scope of their participation, and the confidential handling of information. Subsequently, informed consent and authorisation for the processing of personal data were obtained.

Socio-demographic and clinical characterisation: The following variables were recorded: first and last names, identification, profession, gender, and age (years). In addition, relevant clinical history was documented, such as previous diagnosis of diabetes mellitus (Yes/No), smoking habit (Yes/No), and pharmacological treatment for high blood pressure (HBP) (Yes/No). Blood pressure was measured and recorded as systolic blood pressure (SBP, mmHg) and diastolic blood pressure (DBP, mmHg), and pulse pressure was calculated [44].

#### 2.2.3. Anthropometric and Body Composition Assessment

Measurements of height (m) and body weight (kg) were taken from which Body Mass Index (BMI) was calculated using the formula weight/height^2^ (kg/m^2^). Body composition indicators were also obtained, including body fat percentage, muscle mass percentage, visceral fat index and body water percentage [45,46,47]. These variables enabled a comprehensive characterisation of the nutritional and physical status of the participants.

#### 2.2.4. Biochemical Assessment

A venous blood sample was taken to determine the lipid profile, which included total cholesterol, HDL cholesterol (high-density lipoproteins), LDL cholesterol (low-density lipoproteins), triglycerides, and the total cholesterol/HDL ratio (TC/HDL).

#### 2.2.5. Physical Activity

The physical activity level was assessed through self-reporting and classified into three categories:

Weekly exercise: regular exercise at least once a week.

Monthly exercise: sporadic exercise during the month.

Occasional or infrequent exercise: predominantly sedentary behaviour.

### 2.3. Statistical Analysis

A descriptive and bivariate statistical analysis was performed. Quantitative variables were expressed as means and standard deviations. Qualitative variables were presented as absolute frequencies.

The statistical framework utilised a factorial analysis model (ANOVA) to assess the main effects and the interaction term between age and exercise frequency on pulse pressure. Age was treated as a categorical variable divided into seven average ranges (16–77 years), while exercise frequency was categorised into three levels: weekly, monthly, and occasional.

Data dispersion was represented by error bars corresponding to the standard error of the mean (SEM), facilitating the identification of regions with greater inter-individual variability. All calculations were performed at a 95% confidence level (α = 0.05). The coefficient of variation (CV) is reported for the purpose of identifying the variability of the data set. InfoStat V2020 (Cordoba, Argentina, 2020) statistical software is used to generate interaction graphs that allow the visualisation of deviations from linearity and points of convergence.

## 3. Results

### Anthropometric and Body Composition Results

Once the inclusion protocols have been accepted, participants begin the cardiovascular screening. Table 2 shows the results obtained when taking into account weekly physical activity frequency, female gender, and average age range.

The results indicate that the female cohort with weekly physical activity reveals a gradual physiological transition correlated with advancing age. As the average age increases from 17.95 to 44.6 years, there is a progressive increase in Body Mass Index (BMI), from 22.09 to 28.79 kg/m^2^, and a fourfold increase in the percentage of visceral fat (2.41% to 10.83%). Despite this increase in body composition indicators and a slight increase in diastolic blood pressure (DBP), pulse pressure (PP) remains remarkably stable, ranging from 44.17 to 46.55 mmHg. It is shown that weekly physical activity plays an important modulating role in haemodynamic stability in the face of ageing.

Table 3 below shows the results obtained when taking into account the monthly frequency of physical activity, female gender, and average age range.

Monthly physical activity is identified as having a different cardiovascular dynamic to that observed in the weekly group, characterised by greater variability in metabolic and haemodynamic indicators. Although pulse pressure remains stable between the ages of 17.95 and 62.67 (51.00 to 6.00 mmHg), a progressive deterioration in the lipid profile is observed. Total cholesterol increases significantly in late maturity, reaching 282.00 mg/dL at 62.67 years of age, which correlates with an increase in triglycerides (208.00 mg/dL), LDL cholesterol (183.50 mg/dL) and a reduction in HDL cholesterol (76.00 to 57.00 mg/dL).

Table 4 below shows the results obtained when taking into account occasional or infrequent physical activity, female gender, and average age range.

It is observed that women who engage in occasional physical activity exhibit the profile of greater cardiovascular vulnerability, characterised by an unstable haemodynamic response and a direct correlation between a sedentary lifestyle and arterial stiffness. Although a temporary reduction in pulse pressure is observed around the age of 44.6 (47.33 ± 3.79 mmHg), this indicator rises sharply in old age, reaching a critical value of 75.00 mmHg at the age of 77. This phenomenon coincides with a sustained increase in systolic blood pressure (SBP), which rises from 138.00 to 155.00 mmHg, indicating isolated systolic hypertension typical of ageing without physical observed associations with haemodynamic stability.

From a metabolic perspective, lack of exercise is associated with a Body Mass Index (BMI) that starts at obesity levels (38.15 kg/m^2^) and a considerable visceral fat load (18.25%). Unlike the more active groups, the group with occasional physical activity or exercise shows an inability to regulate LDL cholesterol, which remains high (170.50 mg/dL at age 62).

Table 5 below shows the results obtained when taking into account weekly frequency of physical activity, male gender, and average age range.

It has been observed that in males, weekly physical activity has a sustained protective effect on arterial distensibility despite advancing age and an increase in age-related metabolic risk markers. In the age range of 17.95 to 44.6 years, a progressive increase in risk indicators is observed: total cholesterol rises from 161.36 ± 44.10 to 212.50 ± 22.17 mg/dL and LDL rises from 99.55 to 132.25 mg/dL. Likewise, Body Mass Index (BMI) shows an upward trend, stabilising at around 25 kg/m^2^ in adulthood. Despite this increasing lipid and metabolic profile, pulse pressure (PP) shows remarkable resilience, remaining at optimal levels of 51.45 to 44.00 mmHg. From the above, the frequency of weekly physical activity in men acts as a protective factor that is associated with the impact of increased triglycerides and cholesterol.

Table 6 below shows the results obtained when taking into account monthly frequency of physical activity, male gender, and average age range.

It is observed that monthly physical activity reveals a cardiovascular response characterised by marked fluctuations in haemodynamic efficiency and a progressive deterioration in metabolic profile. Unlike the weekly group, men who engage in monthly physical activity or exercise show unstable pulse pressure (PP); although a decrease is recorded at 44.6 years (33.00 mmHg), it rises again at 62.67 years, reaching 51.33 ± 10.69 mmHg.

From a metabolic perspective, monthly testing is insufficient to contain the risk associated with increasing age. A critical increase in triglyceride levels is observed, rising from 51.00 mg/dL in early youth to a peak of 292.00 mg/dL in maturity (44.6 years), far exceeding the reference ranges. Likewise, total cholesterol shows a sustained upward trend, reaching 203.57 mg/dL at age 54.

Table 7 below shows the results obtained when taking into account occasional or infrequent physical activity, male gender, and average age range.

Occasional physical activity in men is linked to a high cardiovascular risk profile, characterised by an unstable haemodynamic response and a direct correlation between a sedentary lifestyle and arterial stiffness. Unlike groups with higher activity levels, men in this group have significantly elevated systolic blood pressure (SBP) levels from an early age, reaching a critical peak of 170.67 ± 27.14 mmHg at 44.6 years of age.

From a metabolic perspective, lack of exercise is associated with a Body Mass Index (BMI) that consistently remains within the obese range, reaching a maximum of 39.58 kg/m^2^ at 44.6 years of age. This condition is exacerbated by severe visceral fat load (26.33%) and triglyceride levels exceeding 211 mg/dL in older age groups.

## 4. Discussion

This study evaluates the effect of physical activity on conditions that characterise cardiovascular health. It has been identified that occasional physical activity is insufficient to mitigate the impact of vascular and metabolic ageing in men and women, resulting in a loss of arterial distensibility that increases the risk of major cardiovascular events compared to weekly regimes.

**Figure 1:**
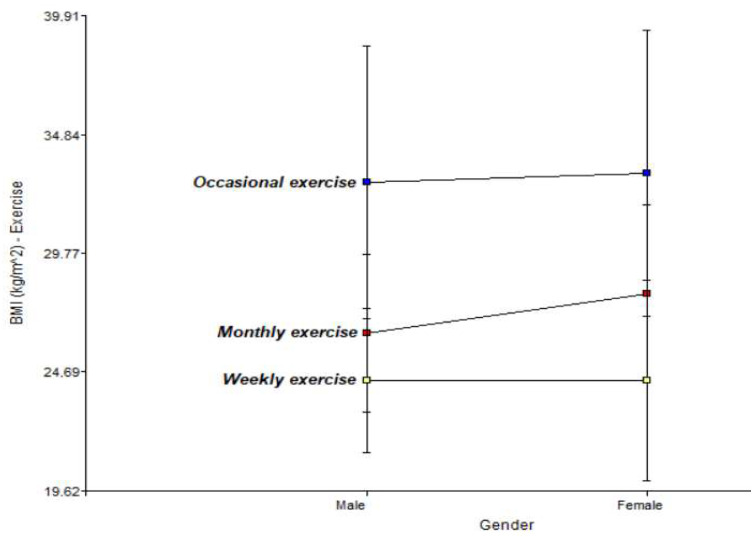
Relationship between physical activity and Body Mass Index (BMI) in men and women.

Figure 1 below shows the two-way interaction graph of the incidence of exercise on the risk factor, Body Mass Index (BMI), for men and women.

With regard to BMI, weekly exercise manages to keep both genders at stable levels (approx. 24.30 kg/m^2^), while occasional exercise raises this indicator above 32 kg/m^2^, correlating directly with an increase in blood pressure.

**Figure 2:**
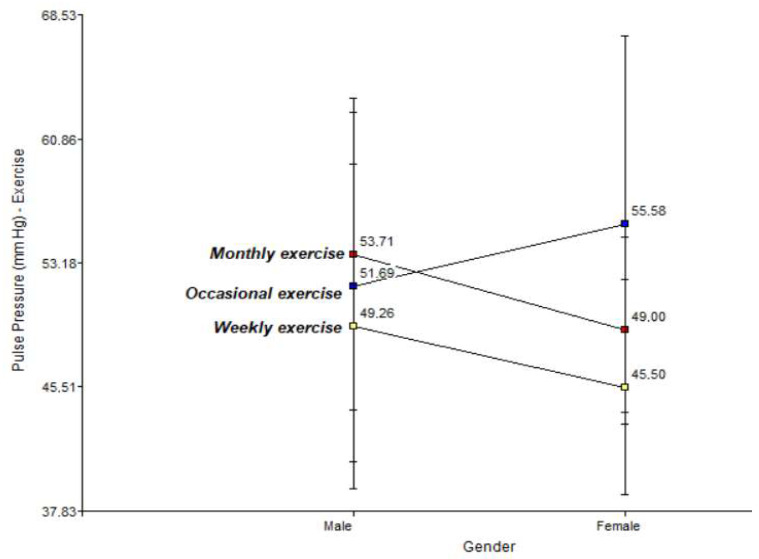
Relationship between physical activity and pulse pressure in men and women.

Figure 2 below illustrates a two-way interaction graph of the incidence of exercise on the risk factor, pulse pressure, for men and women.

The figure shows the interaction by gender, indicating that men have a higher baseline PP in weekly (49.26 mmHg) and monthly (53.71 mmHg) regimens. However, in occasional exercise, women show a more pronounced tendency towards increased PP (55.58 mmHg) than men. The behaviour of pulse pressure (PP) throughout the life cycle shows a non-linear interaction dependent on the frequency of exercise. According to the two-way interaction graph, weekly exercise (yellow line) is confirmed as the most efficient regime, maintaining a stable and decreasing PP over time, reaching the lowest values in the study compared to the monthly and occasional groups.

The results show that weekly physical activity acts as a physiological buffer against vascular ageing. While subjects who exercise occasionally show a pathological increase in pulse pressure and peaks of systolic hypertension related to high levels of visceral fat and BMI, the weekly group manages to dissociate metabolic risk from arterial damage.

**Figure 3:**
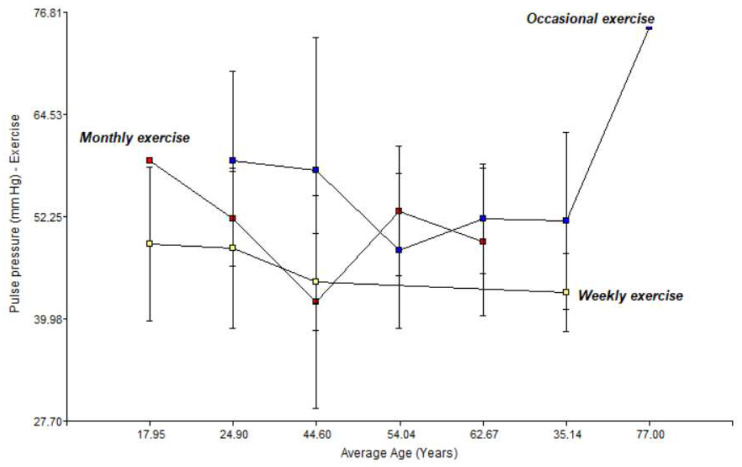
Relationship between physical activity, average age, and pulse pressure in men and women.

Figure 3 below illustrates a two-way interaction graph showing the impact of exercise on the risk factor of pulse pressure in relation to the average age for men and women.

Based on the information, it is demonstrated that the frequency of physical activity is the determining factor in observed associations with haemodynamic stability, the impact of ageing on vascular health. The variable pulse pressure (PP) is a clinical marker of the stiffness of the large arteries. This shows that weekly exercise (yellow line) is the only regimen that maintains a stable and downward trajectory of pulse pressure as age increases. While other groups show erratic fluctuations, subjects with weekly activity manage to maintain their PP in optimal ranges (close to 40–45 mmHg) even in advanced stages.

**Figure 4:**
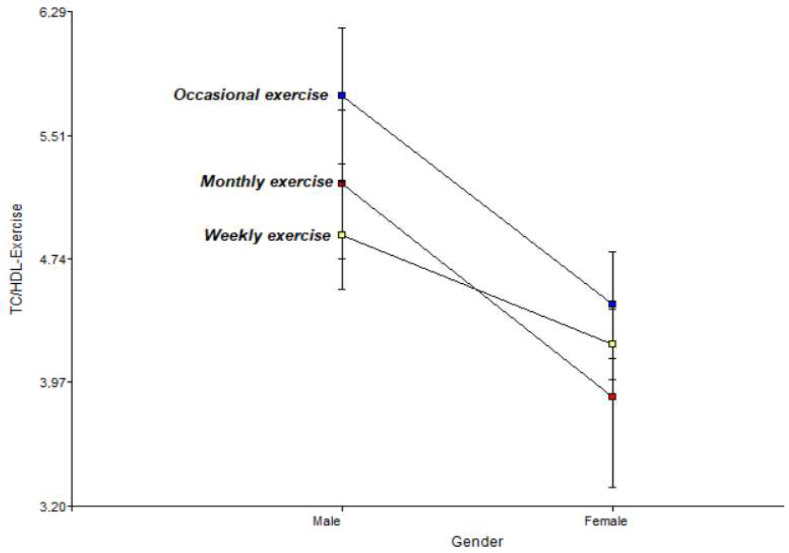
Relationship between physical activity and the total cholesterol/HDL ratio in men and women.

Figure 4 illustrates a two-way interaction graph of the incidence of exercise on the risk factor, total cholesterol/HDL ratio, for men and women.

The graph showing the total cholesterol/HDL ratio reveals a general downward trend from men to women at all activity levels. Men present the highest atherogenic risk under the occasional exercise regime, with an index close to 5.8, which decreases significantly in the weekly exercise group (4.9). The convergence of the lines towards the female side suggests that, metabolically, the women in the study maintain lower and similar lipid risk indices, regardless of whether their activity is monthly or weekly.

**Figure 5:**
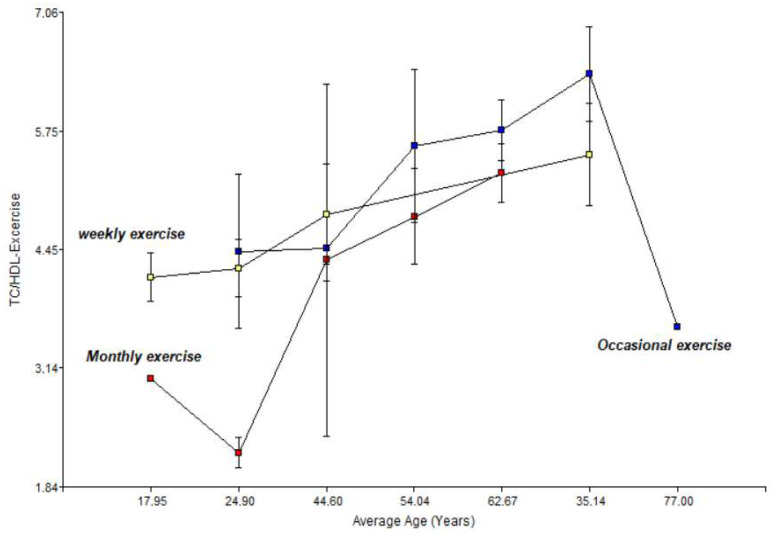
Relationship between physical activity, average age, and total cholesterol/HDL in men and women.

Figure 5 below illustrates a two-way interaction graph showing the impact of exercise on the risk factor, total cholesterol/HDL, in relation to average age.

There is evidence of a male predisposition to higher atherogenic indices. Men who exercise occasionally have the highest risk (5.8), while weekly exercise significantly reduces this index.

The research conducted by [48] shows that men maintain a constant relative strength advantage, although both sexes experience significant deterioration in old age due to sarcopenia. The study addresses the lack of normative data in the frontal and sagittal planes, analysing how physical capacity varies according to gender and age in adults aged 20 to 70. By establishing these benchmarks, other [49] researchers have identified how different types of exercise affect resting testosterone levels in men over 60. The authors analysed 22 studies to determine whether physical training can mitigate the hormonal decline associated with natural ageing. The results indicate that resistance training does not cause significant changes in basal testosterone in this demographic group. It is concluded that, although the effects vary depending on the type of exercise, exercise remains the best non-pharmacological intervention for preserving muscle function.

The study shows that the frequency of physical activity is the determining factor in modulating cardiovascular and metabolic risk throughout ageing. Weekly physical activity is established as the only regimen capable of preserving arterial distensibility, maintaining stable pulse pressure (approx. 44–46 mmHg) and optimal BMI (approx. 24.3 kg/m^2^) in both genders. In contrast, occasional exercise correlates with an increase in vascular stiffness with age [50], reaching critical pulse pressure levels (75 mmHg) at 77 years of age. A marked gender difference in response to inactivity was identified: women are more haemodynamically vulnerable to sporadic exercise, recording the highest pulse pressure in the study (55.58 mmHg). Men, on the other hand, exhibit a more severe atherogenic profile under this same regime, with a TC/HDL ratio of 5.8. Finally, monthly exercise is insufficient to counteract metabolic deterioration, evidenced by triglyceride spikes (292 mg/dL) and marked blood pressure instability. In conclusion, regular weekly exercise is essential for dissociating metabolic risk from systemic vascular damage, ensuring comprehensive protection that cannot be achieved with less frequent or sporadic exercise. Another very important aspect is mental health [51], which is related to improvement, especially in patients diagnosed with CVD.

The authors acknowledge that the non-probabilistic nature of the sample could limit the generalisation of the results to populations with varied socio-demographic contexts and diets. This study did not use a validated instrument (such as the IPAQ or GPAQ) and did not quantify the exact duration (minutes) or intensity (moderate/vigorous) of the sessions, focusing exclusively on the regularity of the habit as a haemodynamic modulator.

## 5. Conclusions

Occasional or monthly exercise is insufficient to counteract age-related cardiovascular and metabolic decline. Sporadic exercise is directly linked to high cardiovascular vulnerability, manifested in a critical increase in arterial stiffness (pulse pressure of up to 75 mmHg in older adults) and an inability to regulate triglyceride and LDL cholesterol levels.

The impact of physical inactivity shows significant gender differences: while women show greater haemodynamic vulnerability to sporadic exercise, recording the highest pulse pressure levels in the study (55.58 mmHg), men develop a more severe atherogenic profile under the same regime, with a risk index (TC/HDL) of 5.8.

In conclusion, regular exercise accompanied by healthy habits promotes cardiovascular health and prevents CVD.

## Figures and Tables

**Table 1:** Average age range and sample percentage.

Age (Years)	Average Age (Years) ± S.D	%*n*
16–19	17.95 ± 1.025	19.82
20–29	24.9 ± 2.10	17.24
30–39	35.14 ± 3.81	12.06
40–49	44.6 ± 2.64	12.93
50–59	54.04 ± 3.42	24.13
60–69	62.67 ± 3.64	12.93
70–79	77 ± 0.00	0.887

±S.D: ±standard deviation; %*n*: percentage of the total sample.

**Table 2:** Anthropometric and biochemical variables in women with weekly exercise frequency.

Average Age (Years)/*n*	17.95/11		24.9/8		35.14/6		44.6/3	
Weekly Exercise Variables	Mean ± S.D	CV%	Mean ± S.D	CV%	Mean ± S.D	CV%	Mean ± S.D	CV%
SBP (systolic blood pressure) (mm Hg)	117.00 ± 12.8	10.95	120.88 ± 13.7	11.37	114.83 ± 8.84	7.70	121.00 ± 7.21	5.96
DBP (diastolic blood pressure) (mm Hg)	70.45 ± 7.71	10.95	75.63 ± 9.93	13.13	70.67 ± 4.41	6.24	76.00 ± 7.00	9.21
Pulse pressure (mmHg)	46.55 ± 9.13	19.61	45.25 ± 5.06	11.19	44.17 ± 5.56	12.60	45.00 ± 1.00	2.22
Treatment (HBP)	0.00 ± 0.00		0.00 ± 0.00		0.00 ± 0.00		0.33 ± 0.58	0.00
Height (m)	1.59 ± 0.08	5.03	1.63 ± 0.05	3.06	1.58 ± 0.03	2.06	1.67 ± 0.17	10.12
Weight (kg)	55.34 ± 8.80	15.90	63.61 ± 10.6	16.74	66.48 ± 9.21	13.85	79.58 ± 10.1	12.78
BMI (kg/m^2^)	22.09 ± 4.00	18.10	24.03 ± 4.13	17.18	26.64 ± 3.20	12.02	28.79 ± 3.01	10.47
% Body fat	8.53 ± 5.51	64.56	13.70 ± 6.63	48.41	15.38 ± 6.73	43.75	23.23 ± 4.80	20.68
% Muscle mass	85.98 ± 15.0	17.53	82.06 ± 6.60	8.05	74.88 ± 15.5	20.75	73.27 ± 4.25	5.80
Visceral fat	2.41 ± 2.48	32.50	4.69 ± 3.79	80.82	5.58 ± 3.54	63.43	10.83 ± 4.31	39.79
% Body water	61.55 ± 3.47	5.65	58.51 ± 3.61	6.17	53.78 ± 8.94	16.63	53.77 ± 2.51	4.67
Total cholesterol (mg/dL)	189.00 ± 55.36	29.29	196.38 ± 57.54	29.30	195.67 ± 72.97	37.29	173.67 ± 20.60	11.86
HDL cholesterol (mg/dL)	52.55 ± 10.53	20.04	49.25 ± 15.88	32.25	39.50 ± 17.74	44.91	38.33 ± 5.13	13.39
Triglycerides (mg/dL)	114.00 ± 39.77	34.89	106.63 ± 42.69	40.04	146.00 ± 80.56	55.18	141.67 ± 62.55	44.15
CT/HDL (mg/dL)	3.64 ± 0.96	26.50	4.10 ± 1.13	27.59	5.21 ± 1.17	22.42	4.56 ± 0.62	13.48
LDL cholesterol (mg/dL)	112.36 ± 47.98	42.70	128.50 ± 42.56	33.12	127.50 ± 52.05	40.83	107.33 ± 23.86	22.23

±S.D: ±standard deviation; CV%: coefficient of variation.

**Table 3:** Anthropometric and biochemical variables in women who exercise monthly.

Average Age (Years)/*n*	17.95/1		54.04/9		62.67/2	
Monthly Exercise Variables	Mean ± S.D	CV%	Mean ± S.D	CV%	Mean ± S.D	CV%
SBP (systolic blood pressure) (mm Hg)	137.00 ± 0.00	0	125.11 ± 11.62	9.29	116.50 ± 7.78	6.68
DBP (diastolic blood pressure) (mm Hg)	86.00 ± 0.00	0	75.67 ± 12.42	16.41	70.50 ± 0.71	1.00
Pulse pressure (mmHg)	51.00 ± 0.00	0	49.44 ± 6.06	12.27	46.00 ± 7.07	15.37
Treatment (HBP)	1.00 ± 0.00	0	0.11 ± 0.33	0.00	0.00 ± 0.00	0.00
Height (m)	1.66 ± 0.00	0	1.64 ± 0.06	3.77	1.58 ± 0.00	0.00
Weight (kg)	77.00 ± 0.00	0	74.78 ± 12.80	17.12	72.63 ± 9.23	12.71
BMI (kg/m^2^)	27.94 ± 0.00	0	27.80 ± 4.22	15.19	29.10 ± 3.70	12.71
% Body fat	22.30 ± 0.00	0	20.44 ± 7.28	35.63	19.60 ± 5.37	27.42
% Muscle mass	74.00 ± 0.00	0	72.63 ± 14.75	20.31	76.45 ± 4.74	6.20
Visceral fat	10.00 ± 0.00	0	9.22 ± 4.72	51.23	7.75 ± 3.89	50.18
% Body water	54.40 ± 0.00	0	55.50 ± 3.72	6.71	55.60 ± 2.26	4.07
Total cholesterol (mg/dL)	182.00 ± 0.00	0	175.67 ± 62.75	35.72	282.00 ± 1.41	0.50
HDL cholesterol (mg/dL)	76.00 ± 0.00	0	52.33 ± 17.04	32.55	57.00 ± 7.07	12,41
Triglycerides (mg/dL)	90.00 ± 0.00	0	169.56 ± 68.25	40.25	208.00 ± 144.25	69.35
CT/HDL (mg/dL)	2.39 ± 0.00	0	3.80 ± 2.15	56.53	4.99 ± 0.64	12.91
LDL cholesterol (mg/dL)	88.00 ± 0.00	0	113.22 ± 34.87	30.80	183.50 ± 20.51	11.17

±S.D: ±standard deviation; CV%: coefficient of variation.

**Table 4:** Anthropometric and biochemical variables in women who exercise occasionally.

Average Age (Years)/*n*	24.9/2		44.6/3		54.04/4		62.67/2		77/1	
Occasional Exercise Variables	Mean ± S.D	CV%	Mean ± S.D	CV%	Mean ± S.D	CV%	Mean ± S.D	CV%	Mean ± S.D	CV%
SBP (systolic blood pressure) (mm Hg)	138.00 ± 8.49	6.15	127.00 ± 6.08	4.79	129.75 ± 14.41	11.10	145.50 ± 13.44	9.23	155.00 ± 0.00	0.00
DBP (diastolic blood pressure) (mm Hg)	71.00 ± 7.07	9.96	79.67 ± 5.51	6.91	77.00 ± 5.77	7.50	93.00 ± 9.90	10.64	80.00 ± 0.00	0.00
Pulse pressure (mmHg)	67.00 ± 15.56	23.22	47.33 ± 3.79	8.00	52.75 ± 10.21	19.36	52.50 ± 3.54	6.73	75.00 ± 0.00	0.00
Treatment (HBP)	0.00 ± 0.0		0.33 ± 0.58		0.25 ± 0.50		0.00 ± 0.0		1.00 ± 0.00	0.00
Height (m)	1.6 ± 0.03	1.77	1.58 ± 0.08	4.76	1.64 ± 0.05	2.92	1.5 ± 0.07	4.56	1.52 ± 0.00	0.00
Weight (kg)	97.53 ± 4.07	4.17	84.00 ± 19.41	23.11	89.30 ± 9.79	10.96	72.10 ± 5.44	7.55	59.15 ± 0.00	0.00
BMI (kg/m^2^)	38.15 ± 2.93	7.69	34.43 ± 10.92	31.71	33.28 ± 3.22	9.68	30.00 ± 0.47	1.56	25.60 ± 0.00	0.00
% Body fat	30.65 ± 1.34	4.38	24.17 ± 9.66	39.99	27.55 ± 4.18	15.18	19.50 ± 3.25	16.68	10.30 ± 0.00	0.00
% Muscle mass	66.70 ± 1.13	1.70	72.43 ± 8.51	11.75	69.43 ± 3.71	5.35	76.50 ± 2.83	3.70	84.60 ± 0.00	0.00
Visceral fat	18.25 ± 1.77	9.69	12.83 ± 8.13	63.34	15.00 ± 4.26	28.41	7.75 ± 2.47	31.93	2.50 ± 0.00	0.00
% Body water	50.90 ± 0.57	1.11	53.67 ± 4.12	7.68	52.25 ± 1.80	3.44	55.65 ± 1.34	2.41	59.50 ± 0.00	0.00
Total cholesterol (mg/dL)	163.00 ± 26.87	16.48	205.33 ± 73.58	35.84	214.00 ± 61.01	28.51	252.00 ± 53.74	21.33	196.00 ± 0.00	0.00
HDL cholesterol (mg/dL)	53.00 ± 7.07	13.34	39.67 ± 10.26	25.87	50.25 ± 11.98	23.85	47.50 ± 14.85	31.26	55.00 ± 0.00	0.00
Triglycerides (mg/dL)	120.50 ± 16.26	13.50	199.00 ± 71.01	35.69	169.25 ± 100.16	59.18	173.00 ± 49.50	28.61	165.00 ± 0.00	0.00
CT/HDL (mg/dL)	3.07 ± 0.10	3.22	5.09 ± 0.49	9.58	4.41 ± 1.44	32.57	5.39 ± 0.55	10.23	3.60 ± 0.00	0.00
LDL cholesterol (mg/dL)	86.50 ± 23.33	26.98	126.33 ± 49.37	39.08	130.50 ± 45.18	34.62	170.50 ± 28.99	17.00	108.00 ± 0.00	0.00

±S.D: ±standard deviation; CV%: coefficient of variation.

**Table 5:** Anthropometric and biochemical variables in men with weekly exercise frequency.

Average Age (Years)/*n*	17.95/11		24.9/5		35.14/3		44.6/4	
Weekly Exercise Variables	Mean ± S.D	CV%	Mean ± S.D	CV%	Mean ± S.D	CV%	Mean ± S.D	CV%
SBP (systolic blood pressure) (mm Hg)	125.00 ± 11.28	9.02	125.20 ± 18.34	14.65	125.67 ± 6.03	4.80	123.00 ± 16.35	13.29
DBP (diastolic blood pressure) (mm Hg)	73.55 ± 8.87	12.06	71.60 ± 10.6	14.80	84.67 ± 6.51	7.68	79.00 ± 11.8	14.94
Pulse pressure (mmHg)	51.45 ± 9.05	17.59	53.60 ± 13.5	25.19	41.00 ± 1.00	2.44	44.00 ± 8.16	18.56
Treatment (HBP)	0.00 ± 0.00		0.00 ± 0.00		0.00 ± 0.00	0.00	0.00 ± 0.00	
Height (m)	1.71 ± 0.08	4.89	1.72 ± 0.07	3.85	1.70 ± 0.05	2.77	1.70 ± 0.06	3.71
Weight (kg)	69.21 ± 14.0	20.33	74.40 ± 10.8	14.58	73.73 ± 3.89	5.27	71.94 ± 4.99	6.93
BMI (kg/m^2^)	23.39 ± 3.40	14.55	25.27 ± 4.10	16.21	25.41 ± 0.61	2.39	24.98 ± 1.42	5.70
% Body fat	16.64 ± 8.86	53.27	20.16 ± 6.56	32.53	20.50 ± 2.17	10.56	19.38 ± 2.91	15.01
% Muscle mass	79.64 ± 8.85	11.11	69.06 ± 14.9	21.69	75.63 ± 1.91	2.53	76.65 ± 2.53	3.30
Visceral fat	6.95 ± 5.32	76.52	8.70 ± 4.48	51.50	8.33 ± 1.53	18.33	7.75 ± 2.10	27.12
% Body water	57.11 ± 4.21	7.37	55.36 ± 2.76	4.98	55.20 ± 0.95	1.73	55.70 ± 1.23	2.21
Total cholesterol (mg/dL)	161.36 ± 44.10	27.33	161.60 ± 43.78	27.09	195.00 ± 18.25	9.36	212.50 ± 22.17	10.43
HDL cholesterol (mg/dL)	33.91 ± 8.61	25.38	37.8 ± 12.32	32.58	37.0 ± 16.09	43.50	46.7 ± 17.29	36.98
Triglycerides (mg/dL)	144.64 ± 107.92	74.62	112.40 ± 83.52	74.31	155.33 ± 54.08	34.81	169.00 ± 53.94	31.92
CT/HDL (mg/dL)	4.70 ± 1.26	26.83	4.46 ± 1.24	27.76	6.04 ± 2.70	44.60	5.04 ± 1.95	38.60
LDL cholesterol (mg/dL)	99.55 ± 29.78	29.92	104.60 ± 28.73	27.46	127.33 ± 32.25	25.33	132.25 ± 13.82	10.45

±S.D: ±standard deviation; CV%: coefficient of variation.

**Table 6:** Anthropometric and biochemical variables in men with monthly exercise frequency.

Average Age (Years)/*n*	17.95/1		24.9/2		44.6/1		54.04/7		62.67/3	
Monthly Exercise Variables	Mean ± S.D	CV%	Mean ± S.D	CV%	Mean ± S.D	CV%	Mean ± S.D	CV%	Mean ± S.D	CV%
SBP (systolic blood pressure) (mm Hg)	134.00 ± 0.00	0	128.50 ± 3.54	2.75	112.00 ± 0.00	0.00	146.43 ± 11.39	7.78	131.00 ± 18.36	14.01
DBP (diastolic blood pressure) (mm Hg)	75.00 ± 0.00	0	76.50 ± 9.19	12.02	79.00 ± 0.00	0.00	89.00 ± 13.04	14.65	79.67 ± 13.87	17.41
Pulse pressure (mmHg)	59.00 ± 0.00	0	52.00 ± 5.66	10.88	33.00 ± 0.00	0.00	57.43 ± 7.85	13.67	51.33 ± 10.69	20.83
Treatment (HBP)	0.00 ± 0.0	0	0.00 ± 0.00		0.00 ± 0.00	0.00	0.43 ± 0.5	124.72	0.00 ± 0.00	
Height (m)	1.62 ± 0.0	0	1.73 ± 0.04	2.05	1.81 ± 0.00	0.00	1.71 ± 0.4	2.54	1.65 ± 0.03	1.60
Weight (kg)	61.50 ± 0.0	0	62.63 ± 1.1	1.75	85.95 ± 0.0	0.00	83.8 ± 6.4	7.20	69.1 ± 5.82	8.42
BMI (kg/m^2^)	23.43 ± 0.00	0	21.07 ± 1.23	5.84	26.24 ± 0.00	0.00	28.76 ± 2.38	8.27	25.35 ± 1.30	5.14
% Body fat	12.30 ± 0.00	0	13.20 ± 0.85	6.43	26.50 ± 0.00	0.00	25.41 ± 2.66	10.46	17.53 ± 3.61	20.60
% Muscle mass	82.90 ± 0.0	0	82.10 ± 0.71	0.86	70.40 ± 0.0	0.00	162.10 ± 239.85	147.96	66.70 ± 19.21	28.80
Visceral fat	3.50 ± 0.00	0	3.75 ± 0.35	9.43	13.50 ± 0.00	0.00	12.71 ± 2.53	19.90	6.33 ± 2.36	37.31
% Body water	58.70 ± 0.00	0	58.35 ± 0.35	0.61	52.70 ± 0.00	0.00	53.13 ± 1.12	2.11	56.43 ± 1.53	2.71
Total cholesterol (mg/dL)	124.00 ± 0.00	0	155.50 ± 0.71	0.45	188.00 ± 0.00	0.00	203.57 ± 39.93	19.62	164.67 ± 20.60	12.51
HDL cholesterol (mg/dL)	41.00 ± 0.00	0	70.50 ± 7.78	11.03	30.00 ± 0.00	0.00	35.00 ± 11.27	32.20	30.33 ± 5.13	16.92
Triglycerides (mg/dL)	51.00 ± 0.00	0	76.50 ± 33.23	43.44	292.00 ± 0.00	0.00	198.71 ± 77.38	38.94	159.33 ± 73.21	45.95
CT/HDL (mg/dL)	3.02 ± 0.0	0	2.22 ± 0.23	10.53	6.27 ± 0.00	0.00	6.11 ± 1.18	19.36	5.49 ± 0.81	14.82
LDL cholesterol (mg/dL)	73.00 ± 0.00	0	70.00 ± 0.00	0.00	100.00 ± 0.00	0.00	129.00 ± 38.85	30.11	103.00 ± 20.88	20.27

±S.D: ±standard deviation; CV%: coefficient of variation.

**Table 7:** Anthropometric and biochemical variables in men who exercise occasionally.

Average Age (Years)/*n*	24.9/2		44.6/3		54.04/4		62.67/2		77/1	
Occasional Exercise Variables	Mean ± S.D	CV%	Mean ± S.D	CV%	Mean ± S.D	CV%	Mean ± S.D	CV%	Mean ± S.D	CV%
SBP (systolic blood pressure) (mm Hg)	137.00 ± 2.65	1.93	128.60 ± 9.42	7.33	170.67 ± 27.14	15.90	132.14 ± 7.60	5.75	136.63 ± 13.72	10.04
DBP (diastolic blood pressure) (mm Hg)	83.33 ± 3.51	4.21	76.80 ± 10.50	13.67	102.33 ± 10.21	9.98	88.71 ± 7.65	8.63	84.75 ± 12.71	15.00
Pulse pressure (mmHg)	53.67 ± 1.15	2.15	51.80 ± 10.64	20.54	68.33 ± 17.10	25.02	43.43 ± 5.38	12.39	51.88 ± 7.36	14.18
Treatment (HBP)	0.00 ± 0.0		0.00 ± 0.00		0.00 ± 0.00		0.29 ± 0.4	0.00	0.50 ± 0.53	0.906
Height (m)	1.8 ± 0.12	6.37	1.74 ± 0.05	2.70	1.72 ± 0.06	3.63	1.77 ± 0.1	5.42	1.69 ± 0.04	2.41
Weight (kg)	114.93 ± 14.39	12.52	105.3 ± 18.3	17.38	116.40 ± 10.3	8.85	97.28 ± 25.72	26.44	85.48 ± 12.55	14.68
BMI (kg/m^2^)	35.05 ± 2.25	6.41	34.64 ± 4.52	13.05	39.58 ± 6.13	15.49	30.82 ± 6.44	20.90	29.99 ± 4.99	16.65
% Body fat	35.17 ± 3.25	9.25	32.42 ± 4.30	13.25	35.70 ± 2.35	6.59	28.54 ± 9.48	33.23	25.62 ± 5.09	19.86
% Muscle mass	60.57 ± 6.22	10.27	57.60 ± 14.23	24.70	59.93 ± 4.61	7.68	55.41 ± 21.74	39.23	58.93 ± 15.76	26.74
Visceral fat	25.67 ± 6.17	24.04	21.60 ± 7.66	35.46	26.33 ± 4.25	16.15	18.29 ± 10.78	58.94	13.38 ± 5.28	39.45
% Body water	49.00 ± 1.37	2.81	50.16 ± 1.84	3.68	48.77 ± 1.00	2.05	51.77 ± 4.05	7.83	49.58 ± 10.00	20.18
Total cholesterol (mg/dL)	153.67 ± 21.01	13.67	175.40 ± 45.41	25.89	169.67 ± 29.02	17.11	178.43 ± 37.33	20.92	188.50 ± 33.67	17.86
HDL cholesterol (mg/dL)	31.00 ± 8.89	28.67	27.60 ± 5.32	19.27	44.33 ± 2.31	5.21	32.43 ± 8.70	26.82	34.00 ± 11.84	34.84
Triglycerides (mg/dL)	169.67 ± 122.46	72.18	217.20 ± 84.72	39.01	168.33 ± 54.04	32.10	194.43 ± 57.77	29.71	211.75 ± 72.81	34.39
CT/HDL (mg/dL)	5.33 ± 2.03	38.05	6.37 ± 1.16	18.17	3.83 ± 0.69	18.02	6.23 ± 3.61	57.89	5.85 ± 1.14	19.55
LDL cholesterol (mg/dL)	89.00 ± 28.84	32.41	104.60 ± 39.90	38.15	92.00 ± 31.00	33.70	108.29 ± 42.04	38.82	113.38 ± 17.62	15.54

±S.D: ±standard deviation; CV%: coefficient of variation.

## Data Availability

The data presented in this study is available on request from the corresponding author. The data is not publicly available due to ethical restrictions.
